# A signaling pathway-driven bioinformatics pipeline for predicting therapeutics against emerging infectious diseases

**DOI:** 10.12688/f1000research.52412.2

**Published:** 2021-08-20

**Authors:** Tiana M. Scott, Sam Jensen, Brett E. Pickett

**Affiliations:** 1 Microbiology and Molecular Biology, Brigham Young University, Provo, Utah, 84602, USA

**Keywords:** bioinformatics, repurposing, coronavirus, SARS-CoV-2, COVID-19, virus, infection, therapeutic, target

## Abstract

**Background: **Severe acute respiratory syndrome coronavirus-2 (SARS-CoV-2), the etiological agent of coronavirus disease-2019 (COVID-19), is a novel Betacoronavirus that was first reported in Wuhan, China in December of 2019. The virus has since caused a worldwide pandemic that highlights the need to quickly identify potential prophylactic or therapeutic treatments that can reduce the signs, symptoms, and/or spread of disease when dealing with a novel infectious agent. To combat this problem, we constructed a computational pipeline that uniquely combines existing tools to predict drugs and biologics that could be repurposed to combat an emerging pathogen.

**Methods:** Our workflow analyzes RNA-sequencing data to determine differentially expressed genes, enriched Gene Ontology (GO) terms, and dysregulated pathways in infected cells, which can then be used to identify US Food and Drug Administration (FDA)-approved drugs that target human proteins within these pathways. We used this pipeline to perform a meta-analysis of RNA-seq data from cells infected with three Betacoronavirus species including severe acute respiratory syndrome coronavirus (SARS-CoV; SARS), Middle East respiratory syndrome coronavirus (MERS-CoV; MERS), and SARS-CoV-2, as well as respiratory syncytial virus and influenza A virus to identify therapeutics that could be used to treat COVID-19.

**Results: **This analysis identified twelve existing drugs, most of which already have FDA-approval, that are predicted to counter the effects of SARS-CoV-2 infection. These results were cross-referenced with interventional clinical trials and other studies in the literature to identify drugs on our list that had previously been identified or used as treatments for COIVD-19 including canakinumab, anakinra, tocilizumab, sarilumab, and baricitinib.

**Conclusions: **While the results reported here are specific to Betacoronaviruses, such as SARS-CoV-2, our bioinformatics pipeline can be used to quickly identify candidate therapeutics for future emerging infectious diseases.

## Introduction

Coronaviruses consist of a lipid envelope that contains a single-stranded positive-sense RNA genome that is approximately 30 kilobases in length. Prior to 2019, six human coronavirus species had been discovered including *HCoV-229E*, *HCoV-NL63*, *HCoV-HKU1*, *HCoV-OC43*, *SARS-CoV*, and *MERS-CoV*. Four of these coronavirus species are endemic in humans and typically cause mild respiratory tract infections that present with cold-like symptoms but can cause more severe symptoms in immunocompromised individuals or infants ^1, 2^. Two of these four endemic virus species are members of the *Alphacoronavirus* genus ( *HCoV-229E* and *HCoV-NL63*), while the other two species are members of the *Betacoronavirus* genus ( *HCoV-HKU1* and *HCoV-OC43*). The remaining two novel human coronavirus species discovered during this time are severe acute respiratory syndrome coronavirus ( *SARS-CoV*; SARS) and Middle East respiratory syndrome coronavirus ( *MERS-CoV*; MERS), which were emergent Betacoronaviruses responsible for epidemics in 2003 and 2012 respectively ^1, 3^. Human coronaviruses generally emerge from other animal hosts such as bats or mice, and typically pass through an intermediate host (e.g. civet cats for SARS and dromedary camels for MERS) before infecting a human host ^1, 3^.

In December 2019, a novel coronavirus was reported in Wuhan city, Hubei province, China and has since been named severe acute respiratory syndrome coronavirus-2 (SARS-CoV-2). SARS-CoV-2 is highly similar to other SARS-like viruses that have been isolated from bats previously, especially BatCoV RaTG13 with which it shares 96.3% identity ^4, 5^. Initial infections with SARS-CoV-2 were traced back to the Huanan Seafood Wholesale Market and likely infected humans via a pangolin intermediate ^6^. The global COVID-19 pandemic, as of February 14, 2021, has resulted in over 108 million cases and over 2.3 million deaths worldwide. Approximately 7.6 million of the cases and over 500,000 deaths have occurred in the United States of America ^7^.

Common symptoms of COVID-19 include fever, dry cough, dyspnea, sore throat, myalgia, fatigue, and in some cases diarrhea ^4, 5, 8– 11^. SARS-CoV-2 is spread through aerosols, droplets, direct contact between people, and fomites ^4, 12^. Other studies suggest a fecal-oral transmission route is possible due to the presence of SARS-CoV-2 in stool samples of infected patients ^12, 13^. SARS-CoV-2 infects cells by binding to the membrane-associated Angiotensin-converting enzyme 2 (ACE2) receptor, which generally plays a role in the renin-angiotensin-aldosterone system to regulate blood pressure and fluid balance in the body ^14, 15^. ACE2 receptors are known to be expressed in lung, renal, cardiac, vascular, intestinal, and placental tissues ^14, 15^.

Both the widespread effects of COVID-19 and the initial highly susceptible population emphasized the need to identify potential drug treatments for emerging diseases--particularly before vaccines become available. The aim of many recent and ongoing clinical trials is to quantify the efficacy of various therapeutics for COVID-19 ^16^. Many vaccines are in various stages of preclinical (at least 139) or clinical (at least 25) development ^17^, as well as some that have gained emergency use authorization by the US Food and Drug Administration (FDA).

The COVID-19 pandemic, caused by the SARS-CoV-2 virus, has highlighted the need to quickly and accurately identify therapeutics that can be repurposed to combat the signs, symptoms, and spread of disease. One method for predicting potential therapeutics is to identify host pathways that are dysregulated by infections and then find existing drugs that target those pathways. Programs to perform this analysis include DrugThatGene and GPSnet, which have both been used to identify potential drugs to target cancers ^18, 19^. The aim of this study was to construct a bioinformatics workflow that uniquely combines existing tools, databases, and programming libraries with custom scripts to predict potential human therapeutic targets for multiple members of the *Betacoronavirus* genus, which includes the *SARS-CoV*, *SARS-CoV-2*, and *MERS-CoV* species. Our unique combination of tools consists of a consistent and robust RNA-seq preprocessing workflow as well as an intracellular signaling pathway perturbation method that enables us to account for the role of protein-protein interaction networks instead of merely enriching for differentially expressed genes. We then applied this workflow to a use case involving a meta-analysis of coronaviruses and other respiratory viruses. This workflow first performs an analysis of human genes and significant signaling pathways that play a role in infection and pathogenesis. The pathway information is then used to predict relevant human drug targets and the associated small molecules or biologics that bind to the target of interest.

The rationale for identifying drug targets from multiple intersecting signaling pathways is based on the theory that a protein which participates in multiple affected pathways during viral infection has a higher likelihood of playing an important role in viral pathogenesis and replication. Targeting one or more of these proteins that act as “key hubs” with a therapeutic would therefore have a higher chance of reducing viral processes and the ensuing disease. Similarly, host proteins that participate in multiple pathways across various related viruses likely represent an evolutionarily conserved host-pathogen interaction that can be therapeutically modulated. We expect virus resistance to these host-based drugs to be relatively infrequent since they target relevant host processes. Our workflow could therefore be applicable not only to improving therapeutic treatment during infection with existing or emerging coronaviruses, such as SARS-CoV-2, but to rapidly identifying potential treatments for pathogens that may emerge in the future.

## Methods

### Included datasets

A search of the Gene Expression Omnibus (GEO) database, hosted at the National Center for Biotechnology Information (NCBI; https://www.ncbi.nlm.nih.gov/geo/) ^20^, was performed in mid-2020 to find RNA-sequencing datasets for various viruses including “MERS”, “SARS”, and “coronavirus”. The corresponding sequencing data for four GEO series were retrieved from the Sequence Read Archive (SRA; https://www.ncbi.nlm.nih.gov/sra) at NCBI ^21^: GSE122876, GSE56192, GSE147507, GSE139516 ^22– 24^. These datasets were generated from cell cultures or patients infected with one of: respiratory syncytial virus (RSV), influenza A virus (IAV), MERS, SARS, and SARS-CoV-2. The SRA files were downloaded and converted to fastq format using version 2.10.1 of the NCBI sratools software package ( https://github.com/ncbi/sra-tools).

### Differential expression analysis

The Automated Reproducible MOdular Workflow for Preprocessing and Differential Analysis of RNA-seq Data (ARMOR) workflow was used to preprocess and analyze the fastq files against the Ensembl reference transcriptome for *Homo sapiens* build GRCh38, release 98 [GCA_000001405.15] ^25^. Briefly, this automated snakemake-based workflow performs quality control of the reads with fastQC ( www.bioinformatics.babraham.ac.uk/projects/fastqc/), trims the adapters and poor-quality regions with TrimGalore! ( https://www.bioinformatics.babraham.ac.uk/projects/trim_galore/), maps & quantifies reads to the human transcriptome using Salmon ^26^, performs differential expression using edgeR ^27^, identifies differential transcript usage with DRIMseq ^28^, and enriches on Gene Ontology terms ^29^ and Hallmark gene sets ^30^ using the Camera algorithm to adjust for inter-gene correlation ^31^. Differential expression was performed by calculating log2 fold-change and the associated p-values from infected samples versus mock-infected samples. The significance threshold for differential gene expression and Gene Ontology enrichment was defined as an false discovery rate (FDR)-corrected p-value < 0.05. Gene Ontology enrichment results from all datasets were then combined to identify shared terms.

### Pathway enrichment analysis

Once the differentially expressed gene lists were constructed, the Ensembl identifiers for these human gene lists were mapped to the corresponding NCBI Entrez Gene identifiers using BioMart and Bioconductor ^32, 33^ prior to pathway enrichment using Signaling Pathway Impact Analysis (SPIA) ^34^. Briefly, this pathway analysis combines robust statistics and bootstrapping to identify enriched pathways from lists of genes by incorporating the directionality of expression. Public pathway databases in the Graphite package in R Bioconductor were used by SPIA for enrichment with those surpassing a Bonferroni-corrected p-value < 0.05 being retained ^35^. These databases include: KEGG ^36^, Reactome ^37^, Panther ^38^, NCI ^39^, and BioCarta. All pathways from each dataset were then compared to identify those that were shared among the various datasets, as well as those that were unique only to infection with SARS-CoV-2.

### Prediction of relevant drug targets

The output from the SPIA pathway enrichment were then used as input for a custom bioinformatics pipeline to identify existing drugs and biologics that could be repurposed to reduce signs, symptoms, and/or replication of coronaviruses. Specifically, this pipeline iteratively 1) retrieves the genes that participate in each statistically significant signaling pathway, 2) maps the gene identifiers to the corresponding UniProt protein identifier ^40^, 3) searches the opentargets resource ( www.opentargets.org ^41^) to identify known drug targets and therapeutics, and 4) generates a table with various attributes of the target and small molecule/biologic treatment. These data were then integrated with the pathway comparisons across the various datasets and analyzed to determine what drugs would affect the pathways dysregulated in the greatest number of viruses and could be used as potential therapeutics for SARS-CoV-2 infection. The results were ranked by how many times a given protein target appeared in the results across the relevant virus taxa. The top ranked small molecules/biologic treatments were analyzed to determine which were predicted to reverse the effects of the viral infection on a given pathway. Manual review of high-ranking hits was then performed to determine the existing indication(s) for each treatment, followed by a literature search to determine which, if any, of the therapeutics identified had previously been used or considered for the treatment of COVID-19. Code for this workflow can be found on GitHub: https://github.com/bpickett/Pathway2Targets.

## Results

### Differential expression analysis

Each of the algorithmic components of our computational workflow was dependent on first calculating differentially expressed (DE) genes for each of the datasets. We consequently began by using the same computational workflow to generate the DE genes from the raw transcriptomic data for human cells infected with one of: RSV, IAV, MERS, SARS, and SARS-CoV-2. Each set of results was specific to the comparison that was performed (e.g. mock-infected vs. infected) and included both log2 fold-change values and FDR-corrected p-values. We then integrated and compared the differential expression results across all virus comparisons to facilitate downstream comparisons.

We began by identifying genes that were either up- or down-regulated across a large number of studies. Specifically, we calculated the number of DE genes (FDR-corrected p-value < 0.05) that significantly changed during infection with influenza A (4205 genes), RSV (3661 genes), MERS (range across studies: 1222-13006 genes), SARS-CoV (range across studies: 2130-5557 genes), or SARS-CoV-2 (range across studies: 427-6933 genes). Out of the 15 studies evaluated, we observed 11 genes that displayed increased expression across 10-13 study comparisons including: TRIM25, C3, NCOA7, PTAFR, TNFAIP3, EIF2AK2, HELZ2, HBEGF, NFKB2, REL and VEGFC. We found an additional 18 genes that were upregulated across 9 of the 15 comparisons including: IFIT2, EGFR, and FOSL1. In contrast, we identified 14 genes that showed decreased expression across 11-12 comparisons including: EIF4H, APH1A, NME2, SEC61B, TUBB4B, CHCHD2, TUBA1C, TP53I3, H2AFZ, PEBP1, HOXB6, TPM2, CBR1, and SIVA1.

We next analyzed the DE lists to identify significant genes that were shared across infections involving only the Betacoronaviruses including SARS-CoV, MERS-CoV, and SARS-CoV-2. This analysis revealed 12 genes that were upregulated in at least nine of thirteen comparisons including: TRIM25, REL, DNASE1, C3, NCOA7, PTAFR, EIF2AK2, HELZ2, NFKB2, ZNF385C, ZC3H12A, and OR7E122P. In contrast, the 10 genes that were found to be downregulated in ten of the thirteen comparisons involving any of the Betacoronaviruses included: CHCHD2, SMIM3, EIF4H, NME2, CBR1, SPARC, DSTN, CDC123, TIMM17A, and PDAP1.

**Figure 1.**
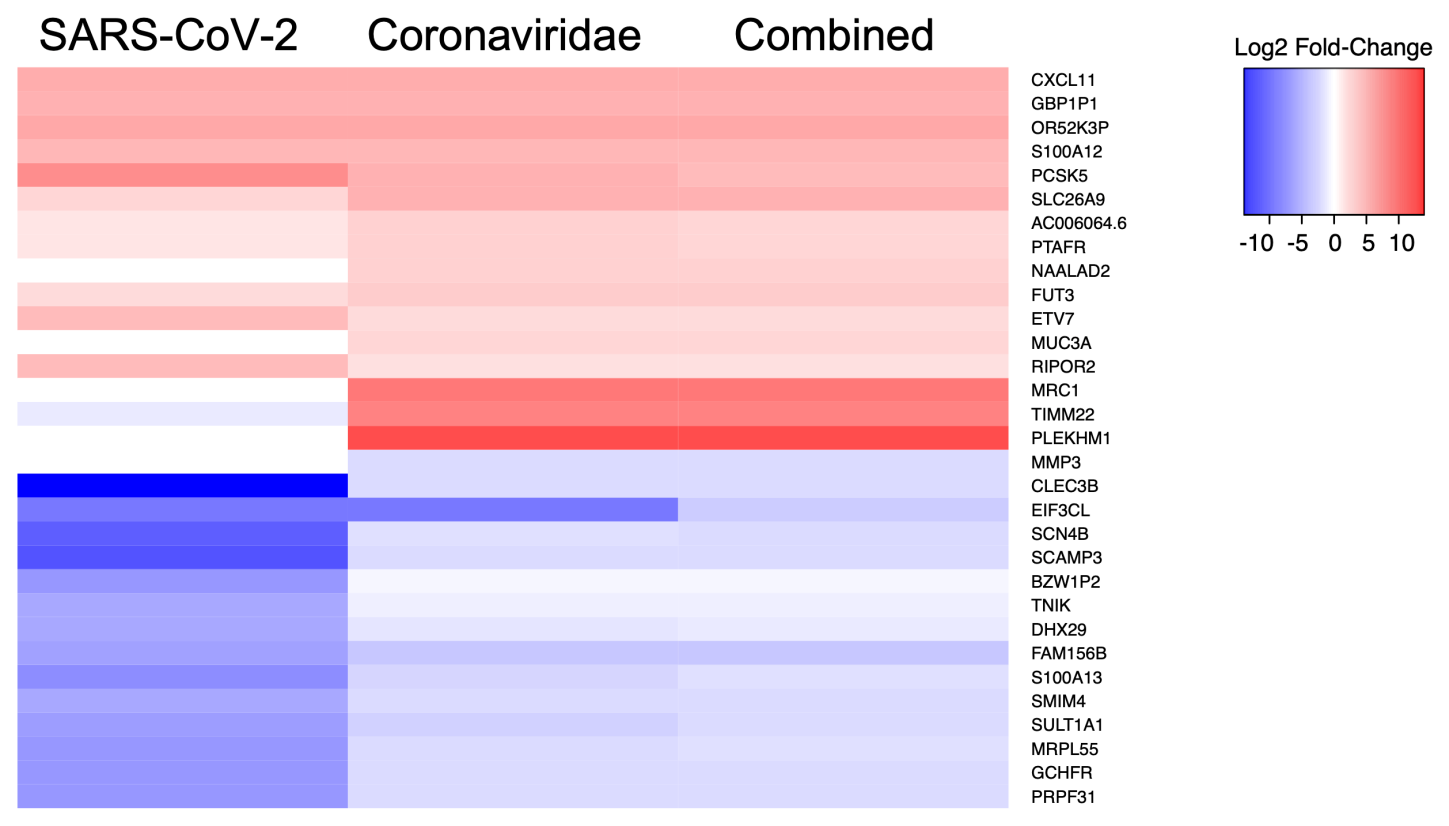
Heatmap of human transcriptional biomarkers associated with infection by various virus taxa. The five genes with the highest and lowest log2 fold-change values for each virus comparison were identified and compared against expression in the other datasets. Squares in red or blue represent genes that are upregulated or downregulated (respectively) in the relevant comparison.

We then performed a similar analysis to identify the five statistically significant DE genes (FDR-corrected p-value < 0.05) that displayed the highest and lowest average fold-change values during infection across all comparisons of SARS-CoV-2, Betacoronaviruses, or all viruses ( Figure 1). This analysis showed that a subset of the genes such as CXCL11, which is induced by interferon and is involved in T-cell signaling, displayed similar fold-change values across multiple comparisons. This finding was somewhat expected since certain genes involved in the host innate immune response are expected to be modulated during virus infection. However, we also observed that genes such as MUC3A, PCSK5, MRC1, and CLEC3B displayed somewhat different average fold-change values across the included comparisons. This observation was also expected given the diverse virus replication requirements and the resulting host intracellular transcriptional response that occurs during infections with human viral pathogens.

### Enrichment of annotated terms

Given the number of DE genes involved in the analysis, manual interpretation of the results would be intractable. We therefore performed an analysis to determine which Gene Ontology (GO) terms and Hallmark gene sets were specifically enriched to better understand which biological processes and molecular functions were represented by the genes in each of the DE gene lists. Overall, we observed a superset of 580 terms that were enriched in any of the included viral infections. The enrichment results for each taxon of virus were then reviewed to identify annotated terms that were shared between multiple types of viral infections, shared amongst coronaviruses, or specific to SARS-CoV-2.

We found several notable terms that were shared among the results obtained from infection of multiple unrelated taxa of viruses. These terms referred to functions such as the host interferon response, regulation of virus response, chemokine activity, and immune cell migration that were positively enriched in both the RSV infection of A549 cells and the SARS-CoV-2 infection of either A549 or NHBE cells. As expected, the statistical significance of these terms supports the important role that the human immune system plays in response to virus infection, which is an expected result and validates the upstream DE gene analysis.

Our method identified no significant GO terms that were shared across all Betacoronavirus comparisons. Interestingly, we found that each coronavirus species had its own set of uniquely enriched terms. In MERS-CoV, “negative regulation of inclusion body assembly” was positively enriched in four of the seven comparisons, while “aggrephagy” and “regulation of nucleotide binding oligomerization domain containing 2 signaling pathway” were positively enriched in three of the seven comparisons. The SARS-CoV samples had very few significantly enriched annotation terms including the “signal peptidase complex” and “cyclin dependent protein serine threonine kinase activator activity” terms that were each negatively enriched in only one of the four SARS-CoV comparisons.

We also identified 290 terms that were uniquely present among the SARS-CoV-2 comparisons, although no significant terms were identified in comparisons involving either the human post-mortem biopsies or the infected Calu-3 cells. The shared significant terms that we identified in the NHBE- and A549-infected cells included “response to chemokine”, “antimicrobial humoral response”, and “humoral immune response.” Enriched terms in the NHBE-infected cells alluded to the role of the inflammatory response, interleukin-1, interleukin-6, neuroinflammation, arachidonic acid binding, and T-cell activation.

### Identification of affected signaling pathways

We then wanted to calculate which intracellular signaling pathways were significantly modulated during viral infection, based on the DE genes identified above. The pathway information used for this analysis was obtained from public databases that manually curate the proteins that participate in conveying a signal from receptors on the cell membrane to transcription factors within the nucleus in order to respond to a stimulus.

We used the results of the differential gene expression analysis as input to the SPIA algorithm. The robust bootstrap-based approach of this method identified 249 pathways that were significantly perturbed across at least one viral comparison. We subsequently analyzed the lists of significantly perturbed pathways for each comparison to determine those that were shared across virus taxa (e.g. influenza A, respiratory syncytial, MERS-CoV, SARS-CoV, and SARS-CoV-2), shared among coronaviruses, and unique to SARS-CoV-2. This analysis revealed a subset of the total number of pathways to be dysregulated across many of the viral taxa studied. These pathways included translation (affected in 12/17 comparisons and 4/5 viral taxa), cytokine-cytokine receptor interaction (modulated in 11/17 comparisons and 4/5 viral taxa), as well as rRNA processing (modulated in 10/17 comparisons and 3/5 viral taxa). We also observed that some of these pathways were predicted to be activated during infection with certain viral taxa in specific cell types, while inhibited in others.

We did not observe any signaling pathways that were significantly and consistently affected during infection by the individual coronavirus species examined. However, we did detect relevant pathways across multiple virus taxa. Interestingly, we found that the direction (i.e. activated or inhibited) was occasionally dependent on virus taxa or timepoint of infection. For example, the cytokine-cytokine receptor interaction pathway was predicted to be activated during infection with RSV, a subset of comparisons involving MERS-CoV, and many SARS-CoV-2 infections; while the same pathway was predicted to be inhibited during infection with SARS-CoV.

**Figure 2.**
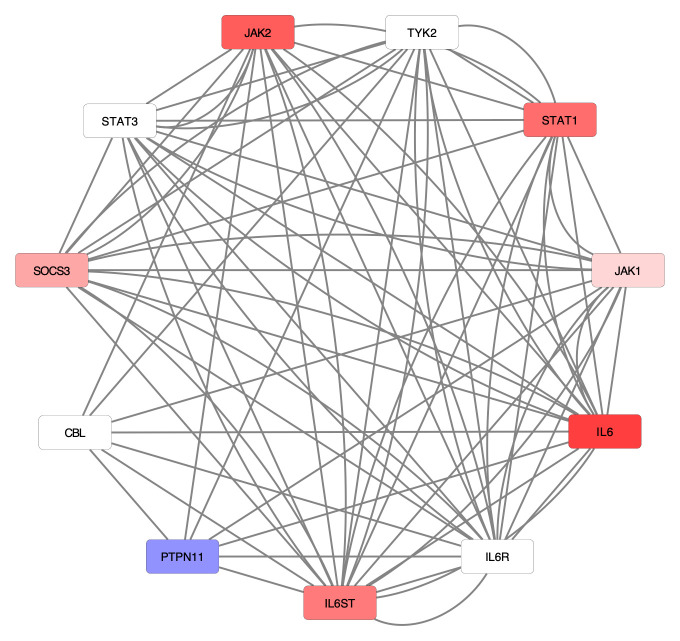
Infection with SARS-CoV-2 shows differential gene expression in Interleukin-6 signaling. This signaling pathway is a component of the larger Cytokine Signaling in Immune System pathway in Reactome. Each node represents a protein in the network, while each edge represents a characterized interaction between the proteins. Higher log2 fold-change values (up-regulation) are represented by increasing saturation of red, while more negative log2-fold-change values (down-regulation) are colored with increased saturation of blue. White nodes indicate no measured log2-fold change.

We predicted a total of 38 pathways that were uniquely affected during SARS-CoV-2 infection ( Table 1). We noted that 35 of these pathways were only found to be significant in one SARS-CoV-2 comparison and included “NF-kB signaling pathway”, “Interleukin-1 signaling”, “IL6-mediated signaling events”, “PI3K-Akt signaling pathway”, “Jak-STAT signaling pathway”, “Apoptosis”, “Complement and coagulation cascades”, and other processes associated with either the immune system or infectious diseases. The remaining three pathways that were predicted as affected during at least two SARS-CoV-2 comparisons were “Cytokine signaling in Immune system”, “Tuberculosis”, and the “Innate immune system”. In short, these findings indicate a set of signaling pathways that are strongly associated with virus infection and/or immune activation in the host, some of which are uniquely detected during SARS-CoV-2 infection. The “Cytokine Signaling in Immune System” pathway is stored in the Reactome database and consists of interferon alpha/beta and gamma signaling, interleukin 1, 2, 6, and 10 signaling, and other components ^37^. To better understand the impact of SARS-CoV-2 on interleukin-6 signaling, we overlaid the differential expression data on a representation of the signaling pathway ( Figure 2). This analysis revealed six of the eleven total proteins in the pathway were upregulated, while another protein was downregulated during SARS-CoV-2 infection.

**Table 1.** Intracellular signaling pathways predicted to be significantly affected during infection with SARS-CoV-2 (GSE147507).

Pathway Name	SARScov2NHBE	SARScov2a549	SARScov2calu3	SARScov2Lung
Tuberculosis			U	U
Innate Immune System			U	U
Cytokine Signaling in Immune system		D	D	
Complement and coagulation cascades	U			
Complement cascade		U		
NF-kappa B signaling pathway			U	
Hepatitis B			U	
Inflammatory bowel disease (IBD)			U	
HTLV-I infection			U	
Jak-STAT signaling pathway			U	
MAPK signaling pathway			U	
Osteoclast differentiation			U	
Apoptosis			U	
Measles			U	
Hepatitis C			U	
Toll-like receptor signaling pathway			U	
Epstein-Barr virus infection			U	
PI3K-Akt signaling pathway			U	
Interleukin-1 signaling			U	
Signaling by Interleukins			U	
Nucleotide-binding domain, leucine rich repeat containing receptor (NLR) signaling pathways			U	
nf-kb signaling pathway			U	
signal transduction through il1r			U	
IL23-mediated signaling events			U	
Angiopoietin receptor Tie2-mediated signaling			U	
IL27-mediated signaling events			U	
IL12-mediated signaling events			U	
IL6-mediated signaling events			U	
Downstream TCR signaling				U
MyD88 dependent cascade initiated on endosome				U
TRAF6 mediated induction of NFkB and MAP kinases upon TLR7/8 or 9 activation				U
Translocation of ZAP-70 to Immunological synapse				U
HDR through Homologous Recombination (HRR)			D	
Class A/1 (Rhodopsin-like receptors)		D		
Peptide ligand-binding receptors		D		
Recognition of DNA damage by PCNA-containing replication complex			D	
Anchoring of the basal body to the plasma membrane			D	
stathmin and breast cancer resistance to antimicrotubule agents			D	

U: upregulated pathway; D: downregulated pathway

### Prediction of relevant drug targets

We next wanted to determine whether any of the significantly affected signaling pathways contained known drug targets that could be modulated to reduce infection, virus replication, and/or clinical signs and symptoms associated with infection by a panel of Betacoronaviruses or by SARS-CoV-2 alone. To do so we cross-referenced the results from our significant pathways analysis with the drug-target information accessible through an application programming interface (API) to the opentargets.org database, which yielded 179 potential human drug targets ( Table 2). We ranked the predicted drugs and their associated targets according to how many coronaviruses shared the same drug target across the various datasets included in our analysis. We performed a separate ranking based on the data obtained solely from the SARS-CoV-2 studies.

**Table 2.** Comparison of predicted human drug targets across multiple datasets.

	GEO Series Identifier														
	GSE122876	GSE139516		GSE56192								GSE147507			
Human Target Symbol	MERS-inf	MERS6	MERS24	MERS high24	MERS low24	MERS low48	MERS high48	SARS low24	SARS high24	SARS low48	SARS high48	SARScov2 NHBE	SARScov2 A549	SARScov2 Calu3	SARScov2 Lung
IFNA1	1	0	1	0	1	0	1	1	1	0	1	1	1	1	1
IFNA4	1	0	1	0	1	0	1	1	1	0	1	1	1	1	1
IFNA5	1	0	1	0	1	0	1	1	1	0	1	1	1	1	1
IFNA6	1	0	1	0	1	0	1	1	1	0	1	1	1	1	1
IFNA7	1	0	1	0	1	0	1	1	1	0	1	1	1	1	1
IFNA8	1	0	1	0	1	0	1	1	1	0	1	1	1	1	1
IFNA10	1	0	1	0	1	0	1	1	1	0	1	1	1	1	1
IFNA13	1	0	1	0	1	0	1	1	1	0	1	1	1	1	1
IFNA14	1	0	1	0	1	0	1	1	1	0	1	1	1	1	1
IL1B	1	0	1	0	1	0	1	1	1	0	1	1	1	1	1
IFNA16	1	0	1	0	1	0	1	1	1	0	1	1	1	1	1
IFNA17	1	0	1	0	1	0	1	1	1	0	1	1	1	1	1
IFNA21	1	0	1	0	1	0	1	1	1	0	1	1	1	1	1
IFNAR1	1	0	1	0	1	0	1	1	1	0	1	1	1	1	1
IFNAR2	1	0	1	0	1	0	1	1	1	0	1	1	1	1	1
IL1A	1	0	1	0	1	0	1	1	1	0	1	1	1	1	1
IL12B	1	0	1	0	1	0	1	1	1	0	1	1	1	1	1
TNFSF13B	1	0	1	0	1	0	1	1	1	0	1	1	0	1	1
CCR4	1	0	1	0	1	0	1	1	1	0	1	1	0	1	1
CCR5	1	0	1	0	1	0	1	1	1	0	1	1	0	1	1
CSF3R	1	0	1	0	1	0	1	1	1	0	1	1	0	1	1
IFNLR1	1	0	1	0	1	0	1	1	1	0	1	1	0	1	1
EPOR	1	0	1	0	1	0	1	1	1	0	1	1	0	1	1
IL17RA	1	0	1	0	1	0	1	1	1	0	1	1	0	1	1
GHR	1	0	1	0	1	0	1	1	1	0	1	1	0	1	1
IL1R1	1	0	1	0	1	0	1	1	1	0	1	1	0	1	1
CXCR4	1	0	1	0	1	0	1	1	1	0	1	1	0	1	1
IL2RA	1	0	1	0	1	0	1	1	1	0	1	1	0	1	1
IL2RB	1	0	1	0	1	0	1	1	1	0	1	1	0	1	1
IL3RA	1	0	1	0	1	0	1	1	1	0	1	1	0	1	1
IL4R	1	0	1	0	1	0	1	1	1	0	1	1	0	1	1
IL5	1	0	1	0	1	0	1	1	1	0	1	1	0	1	1
IL5RA	1	0	1	0	1	0	1	1	1	0	1	1	0	1	1
IL6R	1	0	1	0	1	0	1	1	1	0	1	1	0	1	1
MPL	1	0	1	0	1	0	1	1	1	0	1	1	0	1	1
IL20	1	0	1	0	1	0	1	1	1	0	1	1	0	1	1
TNFRSF4	1	0	1	0	1	0	1	1	1	0	1	1	0	1	1
TNFRSF8	1	0	1	0	1	0	1	1	1	0	1	1	0	1	1
CALM1	0	0	1	0	0	1	1	1	0	1	0	0	0	1	1
HCK	0	0	1	0	1	0	0	0	1	0	1	0	0	1	1
LYN	0	0	1	0	1	0	0	0	1	0	1	0	0	1	1
ROCK1	0	0	1	0	1	0	0	0	1	0	1	0	0	1	1
ROCK2	0	0	1	0	1	0	0	0	1	0	1	0	0	1	1
PDE1A	0	0	1	0	0	1	1	1	0	1	0	0	0	0	0
PDE1C	0	0	1	0	0	1	1	1	0	1	0	0	0	0	0
PDE1B	0	0	1	0	0	1	1	1	0	1	0	0	0	0	0
GSK3B	0	0	0	0	1	0	0	0	1	0	1	0	0	1	1
FGR	0	0	0	0	1	0	0	0	1	0	1	0	0	1	1
GSK3A	0	0	0	0	1	0	0	0	1	0	1	0	0	1	1
ITK	0	0	0	0	1	0	0	0	1	0	1	0	0	1	1
PIK3CD	0	0	0	0	1	0	0	0	1	0	1	0	0	1	1
PIK3CG	0	0	0	0	1	0	0	0	1	0	1	0	0	1	1
PRKCZ	0	0	0	0	1	0	0	0	1	0	1	0	0	1	1
SRC	0	0	0	0	1	0	0	0	1	0	1	0	0	1	1
F2R	0	0	1	0	0	0	0	0	0	0	0	1	0	1	0
MAPK11	0	0	1	0	0	0	0	0	0	0	0	0	0	1	1
JAK1	0	0	0	0	0	0	0	0	0	0	0	0	1	1	1
CDK6	0	0	0	0	0	0	0	0	0	0	1	0	0	1	0
TUBA1B	0	0	1	0	0	0	0	0	0	0	0	0	0	1	0
FDPS	0	0	0	0	0	0	0	0	0	0	0	0	1	1	0
HDAC1	0	0	0	0	0	0	0	0	0	0	1	0	0	1	0
HDAC2	0	0	0	0	0	0	0	0	0	0	1	0	0	1	0
TUBA3E	0	0	1	0	0	0	0	0	0	0	0	0	0	1	0
F2	0	0	1	0	0	0	0	0	0	0	0	1	0	0	0
TUBA4A	0	0	1	0	0	0	0	0	0	0	0	0	0	1	0
TUBA3C	0	0	1	0	0	0	0	0	0	0	0	0	0	1	0
TUBA1A	0	0	1	0	0	0	0	0	0	0	0	0	0	1	0
TLR7	0	0	0	0	0	0	0	0	0	0	0	0	1	1	0
PRKCA	0	0	0	0	0	0	0	0	0	0	0	0	1	1	0
TYK2	0	0	0	0	0	0	0	0	0	0	0	0	1	1	0
TUBA1C	0	0	1	0	0	0	0	0	0	0	0	0	0	1	0
CHRM1	0	0	1	0	0	0	0	0	0	0	0	0	0	1	0
CHRM2	0	0	1	0	0	0	0	0	0	0	0	0	0	1	0
EGFR	0	0	1	0	0	0	0	0	0	0	0	0	0	1	0
ERBB2	0	0	1	0	0	0	0	0	0	0	0	0	0	1	0
ERBB3	0	0	1	0	0	0	0	0	0	0	0	0	0	1	0
BDKRB2	0	0	1	0	0	0	0	0	0	0	0	1	0	0	0
PDGFRB	0	0	1	0	0	0	0	0	0	0	0	0	0	1	0
TLR9	0	0	0	0	0	0	0	0	0	0	0	0	0	1	1
VDR	0	0	0	0	0	0	0	0	0	0	0	0	0	1	1
ABL1	0	0	0	0	0	0	0	0	0	0	1	0	0	0	0
TUBB3	0	0	1	0	0	0	0	0	0	0	0	0	0	0	0
TUBB4A	0	0	1	0	0	0	0	0	0	0	0	0	0	0	0
TUBB4B	0	0	1	0	0	0	0	0	0	0	0	0	0	0	0
RBX1	0	0	0	0	0	0	0	0	0	0	1	0	0	0	0
TUBB8	0	0	1	0	0	0	0	0	0	0	0	0	0	0	0
TUBB2B	0	0	1	0	0	0	0	0	0	0	0	0	0	0	0
MYH7B	0	0	1	0	0	0	0	0	0	0	0	0	0	0	0
TUBB2A	0	0	1	0	0	0	0	0	0	0	0	0	0	0	0
TUBB1	0	0	1	0	0	0	0	0	0	0	0	0	0	0	0
TUBB6	0	0	1	0	0	0	0	0	0	0	0	0	0	0	0
CYSLTR1	0	0	1	0	0	0	0	0	0	0	0	0	0	0	0
CHRM3	0	0	1	0	0	0	0	0	0	0	0	0	0	0	0
CHRM5	0	0	1	0	0	0	0	0	0	0	0	0	0	0	0
ADORA2A	0	0	1	0	0	0	0	0	0	0	0	0	0	0	0
ADORA2B	0	0	1	0	0	0	0	0	0	0	0	0	0	0	0
ADRA1D	0	0	1	0	0	0	0	0	0	0	0	0	0	0	0
ADRA1B	0	0	1	0	0	0	0	0	0	0	0	0	0	0	0
ADRA1A	0	0	1	0	0	0	0	0	0	0	0	0	0	0	0
ADRB1	0	0	1	0	0	0	0	0	0	0	0	0	0	0	0
ADRB2	0	0	1	0	0	0	0	0	0	0	0	0	0	0	0
ADRB3	0	0	1	0	0	0	0	0	0	0	0	0	0	0	0
DRD1	0	0	1	0	0	0	0	0	0	0	0	0	0	0	0
DRD5	0	0	1	0	0	0	0	0	0	0	0	0	0	0	0
AGTR1	0	0	1	0	0	0	0	0	0	0	0	0	0	0	0
EDNRA	0	0	1	0	0	0	0	0	0	0	0	0	0	0	0
F5	0	0	0	0	0	0	0	0	0	0	0	1	0	0	0
EDNRB	0	0	1	0	0	0	0	0	0	0	0	0	0	0	0
F8	0	0	0	0	0	0	0	0	0	0	0	1	0	0	0
PTGS2	0	0	0	0	0	0	0	0	0	0	0	0	0	1	0
F9	0	0	0	0	0	0	0	0	0	0	0	1	0	0	0
BTK	0	0	0	0	0	0	0	0	0	0	0	0	0	1	0
F10	0	0	0	0	0	0	0	0	0	0	0	1	0	0	0
FGB	0	0	0	0	0	0	0	0	0	0	0	1	0	0	0
KLKB1	0	0	0	0	0	0	0	0	0	0	0	1	0	0	0
HRH1	0	0	1	0	0	0	0	0	0	0	0	0	0	0	0
SERPINC1	0	0	0	0	0	0	0	0	0	0	0	1	0	0	0
HRH2	0	0	1	0	0	0	0	0	0	0	0	0	0	0	0
PLAT	0	0	0	0	0	0	0	0	0	0	0	1	0	0	0
HTR2A	0	0	1	0	0	0	0	0	0	0	0	0	0	0	0
PLG	0	0	0	0	0	0	0	0	0	0	0	1	0	0	0
HTR2B	0	0	1	0	0	0	0	0	0	0	0	0	0	0	0
HTR2C	0	0	1	0	0	0	0	0	0	0	0	0	0	0	0
HTR4	0	0	1	0	0	0	0	0	0	0	0	0	0	0	0
HTR5A	0	0	1	0	0	0	0	0	0	0	0	0	0	0	0
HTR6	0	0	1	0	0	0	0	0	0	0	0	0	0	0	0
HTR7	0	0	1	0	0	0	0	0	0	0	0	0	0	0	0
LHCGR	0	0	1	0	0	0	0	0	0	0	0	0	0	0	0
OXTR	0	0	1	0	0	0	0	0	0	0	0	0	0	0	0
CASP12	0	0	0	0	0	0	0	0	0	0	0	0	0	1	0
RRM2	0	0	0	0	0	0	0	0	0	0	1	0	0	0	0
AVPR1A	0	0	1	0	0	0	0	0	0	0	0	0	0	0	0
AVPR1B	0	0	1	0	0	0	0	0	0	0	0	0	0	0	0
PTGER1	0	0	1	0	0	0	0	0	0	0	0	0	0	0	0
PTGER3	0	0	1	0	0	0	0	0	0	0	0	0	0	0	0
PTGFR	0	0	1	0	0	0	0	0	0	0	0	0	0	0	0
TACR2	0	0	1	0	0	0	0	0	0	0	0	0	0	0	0
TACR1	0	0	1	0	0	0	0	0	0	0	0	0	0	0	0
TBXA2R	0	0	1	0	0	0	0	0	0	0	0	0	0	0	0
PRKCG	0	0	0	0	0	0	0	0	0	0	0	0	0	1	0
EPHA2	0	0	0	0	0	0	0	0	0	0	0	0	0	1	0
FGFR3	0	0	0	0	0	0	0	0	0	0	0	0	0	1	0
FGFR4	0	0	0	0	0	0	0	0	0	0	0	0	0	1	0
FLT1	0	0	0	0	0	0	0	0	0	0	0	0	0	1	0
FLT3	0	0	0	0	0	0	0	0	0	0	0	0	0	1	0
FLT4	0	0	0	0	0	0	0	0	0	0	0	0	0	1	0
INSR	0	0	0	0	0	0	0	0	0	0	0	0	0	1	0
KDR	0	0	0	0	0	0	0	0	0	0	0	0	0	1	0
PGF	0	0	0	0	0	0	0	0	0	0	0	0	0	1	0
VEGFA	0	0	0	0	0	0	0	0	0	0	0	0	0	1	0
VEGFB	0	0	0	0	0	0	0	0	0	0	0	0	0	1	0
VEGFC	0	0	0	0	0	0	0	0	0	0	0	0	0	1	0
ITGB3	0	0	0	0	0	0	0	0	0	0	0	0	0	1	0
LCK	0	0	0	0	0	0	0	0	0	0	0	0	0	1	0
PPARG	0	0	0	0	0	0	0	0	0	0	0	0	0	1	0
CALCR	0	0	0	0	0	0	0	0	0	0	0	0	0	1	0
PARP2	0	0	0	0	0	0	0	0	0	0	0	0	0	1	0
PARP3	0	0	0	0	0	0	0	0	0	0	0	0	0	1	0
PARP1	0	0	0	0	0	0	0	0	0	0	0	0	0	1	0
PPARA	0	0	0	0	0	0	0	0	0	0	0	0	0	1	0
RXRA	0	0	0	0	0	0	0	0	0	0	0	0	0	1	0
CD80	0	0	0	0	0	0	0	0	0	0	0	0	0	1	0
CD86	0	0	0	0	0	0	0	0	0	0	0	0	0	1	0
ITGAL	0	0	0	0	0	0	0	0	0	0	0	0	0	1	0
COL1A1	0	0	0	0	0	0	0	0	0	0	0	0	0	1	0
COL1A2	0	0	0	0	0	0	0	0	0	0	0	0	0	1	0
COL4A2	0	0	0	0	0	0	0	0	0	0	0	0	0	1	0
COL4A4	0	0	0	0	0	0	0	0	0	0	0	0	0	1	0
COL4A5	0	0	0	0	0	0	0	0	0	0	0	0	0	1	0
COL4A6	0	0	0	0	0	0	0	0	0	0	0	0	0	1	0
COL6A1	0	0	0	0	0	0	0	0	0	0	0	0	0	1	0
COL6A2	0	0	0	0	0	0	0	0	0	0	0	0	0	1	0
COL6A3	0	0	0	0	0	0	0	0	0	0	0	0	0	1	0
COL6A6	0	0	0	0	0	0	0	0	0	0	0	0	0	1	0
LAMB4	0	0	0	0	0	0	0	0	0	0	0	0	0	1	0
COL6A5	0	0	0	0	0	0	0	0	0	0	0	0	0	1	0
ITGA2B	0	0	0	0	0	0	0	0	0	0	0	0	0	1	0
ITGA4	0	0	0	0	0	0	0	0	0	0	0	0	0	1	0
ITGB7	0	0	0	0	0	0	0	0	0	0	0	0	0	1	0

**KEY: 1 = target was present in comparison, 0 = target was NOT present in comparison

After reviewing the results of this analysis, we identified 38 potential human protein targets to counteract MERS-CoV, SARS-CoV, and/or SARS-CoV-2. Specifically, 38 targets were predicted to be relevant in at least 10 of the 15 coronavirus comparisons ( Table 3). Seventeen of these targets were identified across 11 comparisons and included interferon (IFN)-A, IFN A receptors, IL1A and IL12B. Twenty-one additional targets were identified across 10 comparisons and included members of the tumor necrosis factor (TNF) superfamily, CCR4, CCR5, GHR, CXCR4, IL2R, IL4R, IL5, IL5R, IL6R, MPL, and IL20. We also predicted seven targets that were relevant specifically to SARS-CoV-2 including JAK1, FDPS, TLR7, TLR9, PRKCA, TYK2, and VDR.

**Table 3.** Potential drug candidates for repurposing as antiviral prophylactics or therapeutics.

Target (Gene Symbol)	# Comparisons with Target Detected	Name of Therapeutic	Is Small Molecule	Is Antibody	Is Protein
IFNA1	11	SIFALIMUMAB, RONTALIZUMAB		X, X	
IFNA10	11	SIFALIMUMAB, RONTALIZUMAB		X, X	
IFNA13	11	SIFALIMUMAB, RONTALIZUMAB		X, X	
IFNA14	11	SIFALIMUMAB, RONTALIZUMAB		X, X	
IFNA16	11	SIFALIMUMAB, RONTALIZUMAB		X, X	
IFNA17	11	SIFALIMUMAB, RONTALIZUMAB		X, X	
IFNA21	11	SIFALIMUMAB, RONTALIZUMAB		X, X	
IFNA4	11	SIFALIMUMAB, RONTALIZUMAB		X, X	
IFNA5	11	SIFALIMUMAB, RONTALIZUMAB		X, X	
IFNA6	11	SIFALIMUMAB, RONTALIZUMAB		X, X	
IFNA7	11	SIFALIMUMAB, RONTALIZUMAB		X, X	
IFNA8	11	SIFALIMUMAB, RONTALIZUMAB		X, X	
IFNAR1	11	PEGINTERFERON BETA-1A, PEGINTERFERON ALFA-2A, INTERFERON BETA-1A, INTERFERON BETA-1B,			X, X, X, X
IFNAR2	11	PEGINTERFERON BETA-1A, PEGINTERFERON ALFA-2A, INTERFERON BETA-1A, INTERFERON BETA-1B,			X, X, X, X
IL12B	11	CANAKINUMAB, USTEKINUMAB, BRIAKINUMAB		X, X, X	
IL1A	11	BERMEKIMAB		X	
IL1B	11	CANAKINUMAB		X	
CCR4	10	MOGAMULIZUMAB		X	
CCR5	10	MARAVIROC	X		
CSF3R	10	FILGRASTIM, PEGFILGRASTIM			X, X
CXCR4	10	PLERIXAFOR	X		
EPOR	10	DARBEPOETIN ALFA, PEGINESATIDE			X, X
GHR	10	SOMATROPIN, PEGVISOMANT			X, X
IFNLR1	10	PEGINTERFERON LAMBDA-1A			X, X
IL17RA	10	BRODALUMAB		X	
IL1R1	10	ANAKINRA			X
IL20	10	FLETIKUMAB		X	
IL2RA	10	DACLIZUMAB, DENILEUKIN DIFTITOX		X,	,X
IL2RB	10	ALDESLEUKIN, DENILEUKIN DIFTITOX			X, X
IL3RA	10	TAGRAXOFUSP			X
IL4R	10	DUPILUMAB		X	
IL5	10	MEPOLIZUMAB, RESLIZUMAB		X, X	
IL5RA	10	BENRALIZUMAB		X	
IL6R	10	TOCILIZUMAB		X	
MPL	10	ELTROMBOPAG	X		
TNFRSF4	10	MEDI-6469		X	
TNFRSF8	10	BRENTUXIMAB VEDOTIN		X	
TNFSF13B	10	BELIMUMAB		X	

X: indicates the type of each therapeutic. Drug types against the same target are indicated with commas.

Our analysis predicted twelve existing drugs that are predicted to be useful as repurposed therapeutics against 73.3% of all coronavirus taxa evaluated in this work and 100% of the SARS-CoV-2 comparisons included in our analysis ( Table 4). Eight of these twelve drugs are used to treat common autoimmune disorders such as systemic lupus erythematosus (SLE), Crohn’s disease, and multiple sclerosis (MS). Five of the twelve drugs have been associated or used as antiviral measures largely against hepatitis C virus. Another 27 drugs were predicted to have potential therapeutic activity against 66.7% of all coronavirus infections and 30 were predicted as potential therapeutics against 75% of SARS-CoV-2 infections.

**Table 4.** Comparison of therapeutic drugs and small molecules predicted to be repurposed against SARS-CoV-2.

	GSE122876	GSE139516		GSE56192								GSE147507						Coronavirus Summary				SARS-CoV-2 Summary		
Drug Name	MERS inf	MERS6	MERS24	MERS high24	MERS low24	MERS low48	MERS high48	SARS low24	SARS high24	SARS low48	SARS high48	SARScov2 NHBE	SARScov2 A549	SARScov2 Calu3	SARScov2 Lung	Flu A549	RSV A549	# Positive Comparisons	# Corona	% Corona	Rank (All Corona)	# SARS- CoV-2	% SARS- CoV-2	Rank (SARS- CoV-2)
SIFALIMUMAB	1	0	1	0	1	0	1	1	1	0	1	1	1	1	1	0	1	12	10	66.6666667	1	3	75	1
RONTALIZUMAB	1	0	1	0	1	0	1	1	1	0	1	1	1	1	1	0	1	12	10	66.6666667	1	3	75	1
PEGINTERFERON ALFA-2A	1	0	1	0	1	0	1	1	1	0	1	1	1	1	1	0	1	12	10	66.6666667	1	3	75	1
PEGINTERFERON ALFA-2B	1	0	1	0	1	0	1	1	1	0	1	1	1	1	1	0	1	12	10	66.6666667	1	3	75	1
PEGINTERFERON BETA-1A	1	0	1	0	1	0	1	1	1	0	1	1	1	1	1	0	1	12	10	66.6666667	1	3	75	1
INTERFERON BETA-1A	1	0	1	0	1	0	1	1	1	0	1	1	1	1	1	0	1	12	10	66.6666667	1	3	75	1
INTERFERON BETA-1B	1	0	1	0	1	0	1	1	1	0	1	1	1	1	1	0	1	12	10	66.6666667	1	3	75	1
INTERFERON ALFA-2B	1	0	1	0	1	0	1	1	1	0	1	1	1	1	1	0	1	12	10	66.6666667	1	3	75	1
BERMEKIMAB	1	0	1	0	1	0	1	1	1	0	1	1	1	1	1	0	1	12	10	66.6666667	1	3	75	1
CANAKINUMAB	1	0	1	0	1	0	1	1	1	0	1	1	1	1	1	0	1	12	10	66.6666667	1	3	75	1
USTEKINUMAB	1	0	1	0	1	0	1	1	1	0	1	1	1	1	1	0	1	12	10	66.6666667	1	3	75	1
BRIAKINUMAB	1	0	1	0	1	0	1	1	1	0	1	1	1	1	1	0	1	12	10	66.6666667	1	3	75	1
BELIMUMAB	1	0	1	0	1	0	1	1	1	0	1	1	0	1	1	0	1	11	10	66.6666667	1	3	75	1
MOGAMULIZUMAB	1	0	1	0	1	0	1	1	1	0	1	1	0	1	1	0	1	11	10	66.6666667	1	3	75	1
MARAVIROC	1	0	1	0	1	0	1	1	1	0	1	1	0	1	1	0	1	11	10	66.6666667	1	3	75	1
FILGRASTIM	1	0	1	0	1	0	1	1	1	0	1	1	0	1	1	0	1	11	10	66.6666667	1	3	75	1
PEGFILGRASTIM	1	0	1	0	1	0	1	1	1	0	1	1	0	1	1	0	1	11	10	66.6666667	1	3	75	1
PEGINTERFERON LAMBDA-1A	1	0	1	0	1	0	1	1	1	0	1	1	0	1	1	0	1	11	10	66.6666667	1	3	75	1
DARBEPOETIN ALFA	1	0	1	0	1	0	1	1	1	0	1	1	0	1	1	0	1	11	10	66.6666667	1	3	75	1
PEGINESATIDE	1	0	1	0	1	0	1	1	1	0	1	1	0	1	1	0	1	11	10	66.6666667	1	3	75	1
BRODALUMAB	1	0	1	0	1	0	1	1	1	0	1	1	0	1	1	0	1	11	10	66.6666667	1	3	75	1
SOMATROPIN	1	0	1	0	1	0	1	1	1	0	1	1	0	1	1	0	1	11	10	66.6666667	1	3	75	1
PEGVISOMANT	1	0	1	0	1	0	1	1	1	0	1	1	0	1	1	0	1	11	10	66.6666667	1	3	75	1
ANAKINRA	1	0	1	0	1	0	1	1	1	0	1	1	0	1	1	0	1	11	10	66.6666667	1	3	75	1
DENILEUKIN DIFTITOX	1	0	1	0	1	0	1	1	1	0	1	1	0	1	1	0	1	11	10	66.6666667	1	3	75	1
DACLIZUMAB	1	0	1	0	1	0	1	1	1	0	1	1	0	1	1	0	1	11	10	66.6666667	1	3	75	1
ALDESLEUKIN	1	0	1	0	1	0	1	1	1	0	1	1	0	1	1	0	1	11	10	66.6666667	1	3	75	1
TAGRAXOFUSP	1	0	1	0	1	0	1	1	1	0	1	1	0	1	1	0	1	11	10	66.6666667	1	3	75	1
DUPILUMAB	1	0	1	0	1	0	1	1	1	0	1	1	0	1	1	0	1	11	10	66.6666667	1	3	75	1
MEPOLIZUMAB	1	0	1	0	1	0	1	1	1	0	1	1	0	1	1	0	1	11	10	66.6666667	1	3	75	1
RESLIZUMAB	1	0	1	0	1	0	1	1	1	0	1	1	0	1	1	0	1	11	10	66.6666667	1	3	75	1
BENRALIZUMAB	1	0	1	0	1	0	1	1	1	0	1	1	0	1	1	0	1	11	10	66.6666667	1	3	75	1
TOCILIZUMAB	1	0	1	0	1	0	1	1	1	0	1	1	0	1	1	0	1	11	10	66.6666667	1	3	75	1
SARILUMAB	1	0	1	0	1	0	1	1	1	0	1	1	0	1	1	0	1	11	10	66.6666667	1	3	75	1
ELTROMBOPAG	1	0	1	0	1	0	1	1	1	0	1	1	0	1	1	0	1	11	10	66.6666667	1	3	75	1
FLETIKUMAB	1	0	1	0	1	0	1	1	1	0	1	1	0	1	1	0	1	11	10	66.6666667	1	3	75	1
MEDI-6469	1	0	1	0	1	0	1	1	1	0	1	1	0	1	1	0	1	11	10	66.6666667	1	3	75	1
PLERIXAFOR	1	0	1	0	1	0	1	1	1	0	1	1	0	1	1	0	1	11	10	66.6666667	1	3	75	1
BRENTUXIMAB VEDOTIN	1	0	1	0	1	0	1	1	1	0	1	1	0	1	1	0	1	11	10	66.6666667	1	3	75	1
MIDOSTAURIN	0	0	0	0	1	0	0	0	1	0	1	0	1	1	1	0	1	7	6	40	41	3	75	1
BARICITINIB	0	0	0	0	0	0	0	0	0	0	0	0	1	1	1	0	1	4	3	20	51	3	75	1
HYDROXYCHLOROQUINE	0	0	0	0	0	0	0	0	0	0	0	0	1	1	1	0	1	4	3	20	51	3	75	1
BENZIODARONE	0	0	1	0	0	1	1	1	0	1	0	0	0	1	1	0	0	7	7	46.6666667	40	2	50	43
DASATINIB	0	0	1	0	1	0	0	0	1	0	1	0	0	1	1	0	0	6	6	40	41	2	50	43
NETARSUDIL	0	0	1	0	1	0	0	0	1	0	1	0	0	1	1	0	0	6	6	40	41	2	50	43
LITHIUM CARBONATE	0	0	0	0	1	0	0	0	1	0	1	0	0	1	1	0	0	5	5	33.3333333	44	2	50	43
PAZOPANIB	0	0	0	0	1	0	0	0	1	0	1	0	0	1	1	0	0	5	5	33.3333333	44	2	50	43
IDELALISIB	0	0	0	0	1	0	0	0	1	0	1	0	0	1	1	0	0	5	5	33.3333333	44	2	50	43
DUVELISIB	0	0	0	0	1	0	0	0	1	0	1	0	0	1	1	0	0	5	5	33.3333333	44	2	50	43
REGORAFENIB	0	0	1	0	0	0	0	0	0	0	1	0	0	1	1	0	0	4	4	26.6666667	50	2	50	43
VORAPAXAR	0	0	1	0	0	0	0	0	0	0	0	1	0	1	0	0	0	3	2	13.3333333	53	1	25	58
IMIQUIMOD	0	0	0	0	0	0	0	0	0	0	0	0	1	1	0	0	1	3	2	13.3333333	53	2	50	43
TOFACITINIB	0	0	0	0	0	0	0	0	0	0	0	0	1	1	0	0	1	3	2	13.3333333	53	2	50	43
PARICALCITOL	0	0	0	0	0	0	0	0	0	0	0	0	0	1	1	0	0	2	2	13.3333333	53	2	50	43
CALCIPOTRIENE	0	0	0	0	0	0	0	0	0	0	0	0	0	1	1	0	0	2	2	13.3333333	53	2	50	43
CALCITRIOL	0	0	0	0	0	0	0	0	0	0	0	0	0	1	1	0	0	2	2	13.3333333	53	2	50	43
CHOLECALCIFEROL	0	0	0	0	0	0	0	0	0	0	0	0	0	1	1	0	0	2	2	13.3333333	53	2	50	43
ERGOCALCIFEROL	0	0	0	0	0	0	0	0	0	0	0	0	0	1	1	0	0	2	2	13.3333333	53	2	50	43
ABEMACICLIB	0	0	0	0	0	0	0	0	0	0	1	0	0	1	0	0	0	2	2	13.3333333	53	1	25	58
VORINOSTAT	0	0	0	0	0	0	0	0	0	0	1	0	0	1	0	0	0	2	2	13.3333333	53	1	25	58
PANOBINOSTAT	0	0	0	0	0	0	0	0	0	0	1	0	0	1	0	0	0	2	2	13.3333333	53	1	25	58
ROMIDEPSIN	0	0	0	0	0	0	0	0	0	0	1	0	0	1	0	0	0	2	2	13.3333333	53	1	25	58
VINFLUNINE	0	0	1	0	0	0	0	0	0	0	0	0	0	1	0	0	0	2	2	13.3333333	53	1	25	58
TRASTUZUMAB EMTANSINE	0	0	1	0	0	0	0	0	0	0	0	0	0	1	0	0	0	2	2	13.3333333	53	1	25	58
PACLITAXEL POLIGLUMEX	0	0	1	0	0	0	0	0	0	0	0	0	0	1	0	0	0	2	2	13.3333333	53	1	25	58
BIVALIRUDIN	0	0	1	0	0	0	0	0	0	0	0	1	0	0	0	0	0	2	1	6.66666667	81	0	0	129
DABIGATRAN ETEXILATE	0	0	1	0	0	0	0	0	0	0	0	1	0	0	0	0	0	2	1	6.66666667	81	0	0	129
ARGATROBAN	0	0	1	0	0	0	0	0	0	0	0	1	0	0	0	0	0	2	1	6.66666667	81	0	0	129
DESIRUDIN	0	0	1	0	0	0	0	0	0	0	0	1	0	0	0	0	0	2	1	6.66666667	81	0	0	129
GLYCOPYRRONIUM	0	0	1	0	0	0	0	0	0	0	0	0	0	1	0	0	0	2	2	13.3333333	53	1	25	58
ATROPINE	0	0	1	0	0	0	0	0	0	0	0	0	0	1	0	0	0	2	2	13.3333333	53	1	25	58
BENZTROPINE	0	0	1	0	0	0	0	0	0	0	0	0	0	1	0	0	0	2	2	13.3333333	53	1	25	58
TERODILINE	0	0	1	0	0	0	0	0	0	0	0	0	0	1	0	0	0	2	2	13.3333333	53	1	25	58
TROSPIUM	0	0	1	0	0	0	0	0	0	0	0	0	0	1	0	0	0	2	2	13.3333333	53	1	25	58
SOLIFENACIN	0	0	1	0	0	0	0	0	0	0	0	0	0	1	0	0	0	2	2	13.3333333	53	1	25	58
OXYBUTYNIN	0	0	1	0	0	0	0	0	0	0	0	0	0	1	0	0	0	2	2	13.3333333	53	1	25	58
DARIFENACIN	0	0	1	0	0	0	0	0	0	0	0	0	0	1	0	0	0	2	2	13.3333333	53	1	25	58
TOLTERODINE	0	0	1	0	0	0	0	0	0	0	0	0	0	1	0	0	0	2	2	13.3333333	53	1	25	58
GEFITINIB	0	0	1	0	0	0	0	0	0	0	0	0	0	1	0	0	0	2	2	13.3333333	53	1	25	58
ERLOTINIB	0	0	1	0	0	0	0	0	0	0	0	0	0	1	0	0	0	2	2	13.3333333	53	1	25	58
VANDETANIB	0	0	1	0	0	0	0	0	0	0	0	0	0	1	0	0	0	2	2	13.3333333	53	1	25	58
NINTEDANIB	0	0	1	0	0	0	0	0	0	0	0	0	0	1	0	0	0	2	2	13.3333333	53	1	25	58
ICATIBANT	0	0	1	0	0	0	0	0	0	0	0	1	0	0	0	0	0	2	1	6.66666667	81	0	0	129
RISEDRONIC ACID	0	0	0	0	0	0	0	0	0	0	0	0	1	0	0	0	1	2	1	6.66666667	81	1	25	58
ALENDRONIC ACID	0	0	0	0	0	0	0	0	0	0	0	0	1	0	0	0	1	2	1	6.66666667	81	1	25	58
ZOLEDRONIC ACID	0	0	0	0	0	0	0	0	0	0	0	0	1	0	0	0	1	2	1	6.66666667	81	1	25	58
PAMIDRONIC ACID	0	0	0	0	0	0	0	0	0	0	0	0	1	0	0	0	1	2	1	6.66666667	81	1	25	58
DROTRECOGIN ALFA (ACTIVATED)	0	0	0	0	0	0	0	0	0	0	0	1	0	0	0	0	0	1	0	0	235	0	0	129
EMICIZUMAB	0	0	0	0	0	0	0	0	0	0	0	1	0	0	0	0	0	1	0	0	235	0	0	129
EDOXABAN	0	0	0	0	0	0	0	0	0	0	0	1	0	0	0	0	0	1	0	0	235	0	0	129
RIVAROXABAN	0	0	0	0	0	0	0	0	0	0	0	1	0	0	0	0	0	1	0	0	235	0	0	129
APIXABAN	0	0	0	0	0	0	0	0	0	0	0	1	0	0	0	0	0	1	0	0	235	0	0	129
FIBRINOLYSIN, HUMAN	0	0	0	0	0	0	0	0	0	0	0	1	0	0	0	0	0	1	0	0	235	0	0	129
APROTININ	0	0	0	0	0	0	0	0	0	0	0	1	0	0	0	0	0	1	0	0	235	0	0	129
ECALLANTIDE	0	0	0	0	0	0	0	0	0	0	0	1	0	0	0	0	0	1	0	0	235	0	0	129
LANADELUMAB	0	0	0	0	0	0	0	0	0	0	0	1	0	0	0	0	0	1	0	0	235	0	0	129
HEPARIN SODIUM	0	0	0	0	0	0	0	0	0	0	0	1	0	0	0	0	0	1	0	0	235	0	0	129
ENOXAPARIN SODIUM	0	0	0	0	0	0	0	0	0	0	0	1	0	0	0	0	0	1	0	0	235	0	0	129
TINZAPARIN SODIUM	0	0	0	0	0	0	0	0	0	0	0	1	0	0	0	0	0	1	0	0	235	0	0	129
AMINOCAPROIC ACID	0	0	0	0	0	0	0	0	0	0	0	1	0	0	0	0	0	1	0	0	235	0	0	129
TRANEXAMIC ACID	0	0	0	0	0	0	0	0	0	0	0	1	0	0	0	0	0	1	0	0	235	0	0	129
UROKINASE	0	0	0	0	0	0	0	0	0	0	0	1	0	0	0	0	0	1	0	0	235	0	0	129
ALTEPLASE	0	0	0	0	0	0	0	0	0	0	0	1	0	0	0	0	0	1	0	0	235	0	0	129
ACETAMINOPHEN	0	0	0	0	0	0	0	0	0	0	0	0	0	1	0	0	0	1	1	6.66666667	81	1	25	58
ACECLOFENAC	0	0	0	0	0	0	0	0	0	0	0	0	0	1	0	0	0	1	1	6.66666667	81	1	25	58
ASPIRIN	0	0	0	0	0	0	0	0	0	0	0	0	0	1	0	0	0	1	1	6.66666667	81	1	25	58
IBRUTINIB	0	0	0	0	0	0	0	0	0	0	0	0	0	1	0	0	0	1	1	6.66666667	81	1	25	58
EMRICASAN	0	0	0	0	0	0	0	0	0	0	0	0	0	1	0	0	0	1	1	6.66666667	81	1	25	58
Small molecule	0	0	0	0	0	0	0	0	0	0	0	0	0	1	0	0	0	1	1	6.66666667	81	1	25	58
Protein	0	0	0	0	0	0	0	0	0	0	0	0	0	1	0	0	0	1	1	6.66666667	81	1	25	58
ERDAFITINIB	0	0	0	0	0	0	0	0	0	0	0	0	0	1	0	0	0	1	1	6.66666667	81	1	25	58
SUNITINIB	0	0	0	0	0	0	0	0	0	0	0	0	0	1	0	0	0	1	1	6.66666667	81	1	25	58
LENVATINIB	0	0	0	0	0	0	0	0	0	0	0	0	0	1	0	0	0	1	1	6.66666667	81	1	25	58
SORAFENIB	0	0	0	0	0	0	0	0	0	0	0	0	0	1	0	0	0	1	1	6.66666667	81	1	25	58
AXITINIB	0	0	0	0	0	0	0	0	0	0	0	0	0	1	0	0	0	1	1	6.66666667	81	1	25	58
INSULIN GLARGINE	0	0	0	0	0	0	0	0	0	0	0	0	0	1	0	0	0	1	1	6.66666667	81	1	25	58
INSULIN SUSP ISOPHANE BEEF	0	0	0	0	0	0	0	0	0	0	0	0	0	1	0	0	0	1	1	6.66666667	81	1	25	58
INSULIN GLULISINE	0	0	0	0	0	0	0	0	0	0	0	0	0	1	0	0	0	1	1	6.66666667	81	1	25	58
INSULIN LISPRO	0	0	0	0	0	0	0	0	0	0	0	0	0	1	0	0	0	1	1	6.66666667	81	1	25	58
INSULIN DETEMIR	0	0	0	0	0	0	0	0	0	0	0	0	0	1	0	0	0	1	1	6.66666667	81	1	25	58
INSULIN PORK	0	0	0	0	0	0	0	0	0	0	0	0	0	1	0	0	0	1	1	6.66666667	81	1	25	58
ANLOTINIB	0	0	0	0	0	0	0	0	0	0	0	0	0	1	0	0	0	1	1	6.66666667	81	1	25	58
CABOZANTINIB	0	0	0	0	0	0	0	0	0	0	0	0	0	1	0	0	0	1	1	6.66666667	81	1	25	58
AFLIBERCEPT	0	0	0	0	0	0	0	0	0	0	0	0	0	1	0	0	0	1	1	6.66666667	81	1	25	58
RANIBIZUMAB	0	0	0	0	0	0	0	0	0	0	0	0	0	1	0	0	0	1	1	6.66666667	81	1	25	58
CONBERCEPT	0	0	0	0	0	0	0	0	0	0	0	0	0	1	0	0	0	1	1	6.66666667	81	1	25	58
TIROFIBAN	0	0	0	0	0	0	0	0	0	0	0	0	0	1	0	0	0	1	1	6.66666667	81	1	25	58
PIOGLITAZONE	0	0	0	0	0	0	0	0	0	0	0	0	0	1	0	0	0	1	1	6.66666667	81	1	25	58
MESALAMINE	0	0	0	0	0	0	0	0	0	0	0	0	0	1	0	0	0	1	1	6.66666667	81	1	25	58
ROSIGLITAZONE	0	0	0	0	0	0	0	0	0	0	0	0	0	1	0	0	0	1	1	6.66666667	81	1	25	58
PRAMLINTIDE	0	0	0	0	0	0	0	0	0	0	0	0	0	1	0	0	0	1	1	6.66666667	81	1	25	58
CALCITONIN SALMON RECOMBINANT	0	0	0	0	0	0	0	0	0	0	0	0	0	1	0	0	0	1	1	6.66666667	81	1	25	58
CALCITONIN SALMON	0	0	0	0	0	0	0	0	0	0	0	0	0	1	0	0	0	1	1	6.66666667	81	1	25	58
CALCITONIN	0	0	0	0	0	0	0	0	0	0	0	0	0	1	0	0	0	1	1	6.66666667	81	1	25	58
TALAZOPARIB	0	0	0	0	0	0	0	0	0	0	0	0	0	1	0	0	0	1	1	6.66666667	81	1	25	58
NIRAPARIB	0	0	0	0	0	0	0	0	0	0	0	0	0	1	0	0	0	1	1	6.66666667	81	1	25	58
OLAPARIB	0	0	0	0	0	0	0	0	0	0	0	0	0	1	0	0	0	1	1	6.66666667	81	1	25	58
RUCAPARIB	0	0	0	0	0	0	0	0	0	0	0	0	0	1	0	0	0	1	1	6.66666667	81	1	25	58
VELIPARIB	0	0	0	0	0	0	0	0	0	0	0	0	0	1	0	0	0	1	1	6.66666667	81	1	25	58
FENOFIBRATE	0	0	0	0	0	0	0	0	0	0	0	0	0	1	0	0	0	1	1	6.66666667	81	1	25	58
FENOFIBRIC ACID	0	0	0	0	0	0	0	0	0	0	0	0	0	1	0	0	0	1	1	6.66666667	81	1	25	58
ACITRETIN	0	0	0	0	0	0	0	0	0	0	0	0	0	1	0	0	0	1	1	6.66666667	81	1	25	58
ABATACEPT	0	0	0	0	0	0	0	0	0	0	0	0	0	1	0	0	0	1	1	6.66666667	81	1	25	58
BELATACEPT	0	0	0	0	0	0	0	0	0	0	0	0	0	1	0	0	0	1	1	6.66666667	81	1	25	58
EFALIZUMAB	0	0	0	0	0	0	0	0	0	0	0	0	0	1	0	0	0	1	1	6.66666667	81	1	25	58
COLLAGENASE CLOSTRIDIUM HISTOLYTICUM	0	0	0	0	0	0	0	0	0	0	0	0	0	1	0	0	0	1	1	6.66666667	81	1	25	58
OCRIPLASMIN	0	0	0	0	0	0	0	0	0	0	0	0	0	1	0	0	0	1	1	6.66666667	81	1	25	58
VEDOLIZUMAB	0	0	0	0	0	0	0	0	0	0	0	0	0	1	0	0	0	1	1	6.66666667	81	1	25	58
NATALIZUMAB	0	0	0	0	0	0	0	0	0	0	0	0	0	1	0	0	0	1	1	6.66666667	81	1	25	58
PENTOXIFYLLINE	0	0	1	0	0	1	1	1	0	1	0	0	0	0	0	0	0	5	5	33.3333333	44	0	0	129
DIPYRIDAMOLE	0	0	1	0	0	1	1	1	0	1	0	0	0	0	0	0	0	5	5	33.3333333	44	0	0	129
NILOTINIB	0	0	0	0	0	0	0	0	0	0	1	0	0	0	0	0	0	1	1	6.66666667	81	0	0	129
IMATINIB	0	0	0	0	0	0	0	0	0	0	1	0	0	0	0	0	0	1	1	6.66666667	81	0	0	129
LENALIDOMIDE	0	0	0	0	0	0	0	0	0	0	1	0	0	0	0	0	0	1	1	6.66666667	81	0	0	129
THALIDOMIDE	0	0	0	0	0	0	0	0	0	0	1	0	0	0	0	0	0	1	1	6.66666667	81	0	0	129
HYDROXYUREA	0	0	0	0	0	0	0	0	0	0	1	0	0	0	0	0	0	1	1	6.66666667	81	0	0	129
DOCETAXEL	0	0	1	0	0	0	0	0	0	0	0	0	0	0	0	0	0	1	1	6.66666667	81	0	0	129
CABAZITAXEL	0	0	1	0	0	0	0	0	0	0	0	0	0	0	0	0	0	1	1	6.66666667	81	0	0	129
PACLITAXEL	0	0	1	0	0	0	0	0	0	0	0	0	0	0	0	0	0	1	1	6.66666667	81	0	0	129
COLCHICINE	0	0	1	0	0	0	0	0	0	0	0	0	0	0	0	0	0	1	1	6.66666667	81	0	0	129
VINCRISTINE	0	0	1	0	0	0	0	0	0	0	0	0	0	0	0	0	0	1	1	6.66666667	81	0	0	129
IXABEPILONE	0	0	1	0	0	0	0	0	0	0	0	0	0	0	0	0	0	1	1	6.66666667	81	0	0	129
VINORELBINE	0	0	1	0	0	0	0	0	0	0	0	0	0	0	0	0	0	1	1	6.66666667	81	0	0	129
VINBLASTINE	0	0	1	0	0	0	0	0	0	0	0	0	0	0	0	0	0	1	1	6.66666667	81	0	0	129
OMECAMTIV MECARBIL	0	0	1	0	0	0	0	0	0	0	0	0	0	0	0	0	0	1	1	6.66666667	81	0	0	129
MONTELUKAST	0	0	1	0	0	0	0	0	0	0	0	0	0	0	0	0	0	1	1	6.66666667	81	0	0	129
ZAFIRLUKAST	0	0	1	0	0	0	0	0	0	0	0	0	0	0	0	0	0	1	1	6.66666667	81	0	0	129
IPRATROPIUM	0	0	1	0	0	0	0	0	0	0	0	0	0	0	0	0	0	1	1	6.66666667	81	0	0	129
TROPICAMIDE	0	0	1	0	0	0	0	0	0	0	0	0	0	0	0	0	0	1	1	6.66666667	81	0	0	129
TIOTROPIUM	0	0	1	0	0	0	0	0	0	0	0	0	0	0	0	0	0	1	1	6.66666667	81	0	0	129
UMECLIDINIUM	0	0	1	0	0	0	0	0	0	0	0	0	0	0	0	0	0	1	1	6.66666667	81	0	0	129
FESOTERODINE	0	0	1	0	0	0	0	0	0	0	0	0	0	0	0	0	0	1	1	6.66666667	81	0	0	129
CAFFEINE	0	0	1	0	0	0	0	0	0	0	0	0	0	0	0	0	0	1	1	6.66666667	81	0	0	129
THEOPHYLLINE	0	0	1	0	0	0	0	0	0	0	0	0	0	0	0	0	0	1	1	6.66666667	81	0	0	129
ADENOSINE	0	0	1	0	0	0	0	0	0	0	0	0	0	0	0	0	0	1	1	6.66666667	81	0	0	129
CARVEDILOL	0	0	1	0	0	0	0	0	0	0	0	0	0	0	0	0	0	1	1	6.66666667	81	0	0	129
NOREPINEPHRINE	0	0	1	0	0	0	0	0	0	0	0	0	0	0	0	0	0	1	1	6.66666667	81	0	0	129
EPINEPHRINE	0	0	1	0	0	0	0	0	0	0	0	0	0	0	0	0	0	1	1	6.66666667	81	0	0	129
NAPHAZOLINE	0	0	1	0	0	0	0	0	0	0	0	0	0	0	0	0	0	1	1	6.66666667	81	0	0	129
TAMSULOSIN	0	0	1	0	0	0	0	0	0	0	0	0	0	0	0	0	0	1	1	6.66666667	81	0	0	129
DOXAZOSIN	0	0	1	0	0	0	0	0	0	0	0	0	0	0	0	0	0	1	1	6.66666667	81	0	0	129
ERGOTAMINE	0	0	1	0	0	0	0	0	0	0	0	0	0	0	0	0	0	1	1	6.66666667	81	0	0	129
ALFUZOSIN	0	0	1	0	0	0	0	0	0	0	0	0	0	0	0	0	0	1	1	6.66666667	81	0	0	129
METARAMINOL	0	0	1	0	0	0	0	0	0	0	0	0	0	0	0	0	0	1	1	6.66666667	81	0	0	129
PRAZOSIN	0	0	1	0	0	0	0	0	0	0	0	0	0	0	0	0	0	1	1	6.66666667	81	0	0	129
SERTINDOLE	0	0	1	0	0	0	0	0	0	0	0	0	0	0	0	0	0	1	1	6.66666667	81	0	0	129
ATENOLOL	0	0	1	0	0	0	0	0	0	0	0	0	0	0	0	0	0	1	1	6.66666667	81	0	0	129
METOPROLOL	0	0	1	0	0	0	0	0	0	0	0	0	0	0	0	0	0	1	1	6.66666667	81	0	0	129
BISOPROLOL	0	0	1	0	0	0	0	0	0	0	0	0	0	0	0	0	0	1	1	6.66666667	81	0	0	129
SOTALOL	0	0	1	0	0	0	0	0	0	0	0	0	0	0	0	0	0	1	1	6.66666667	81	0	0	129
ALBUTEROL	0	0	1	0	0	0	0	0	0	0	0	0	0	0	0	0	0	1	1	6.66666667	81	0	0	129
TERBUTALINE	0	0	1	0	0	0	0	0	0	0	0	0	0	0	0	0	0	1	1	6.66666667	81	0	0	129
FORMOTEROL	0	0	1	0	0	0	0	0	0	0	0	0	0	0	0	0	0	1	1	6.66666667	81	0	0	129
LABETALOL	0	0	1	0	0	0	0	0	0	0	0	0	0	0	0	0	0	1	1	6.66666667	81	0	0	129
PROPRANOLOL	0	0	1	0	0	0	0	0	0	0	0	0	0	0	0	0	0	1	1	6.66666667	81	0	0	129
LEVOSALBUTAMOL	0	0	1	0	0	0	0	0	0	0	0	0	0	0	0	0	0	1	1	6.66666667	81	0	0	129
DROXIDOPA	0	0	1	0	0	0	0	0	0	0	0	0	0	0	0	0	0	1	1	6.66666667	81	0	0	129
METHYLERGONOVINE	0	0	1	0	0	0	0	0	0	0	0	0	0	0	0	0	0	1	1	6.66666667	81	0	0	129
PIMOZIDE	0	0	1	0	0	0	0	0	0	0	0	0	0	0	0	0	0	1	1	6.66666667	81	0	0	129
ERGOLOID	0	0	1	0	0	0	0	0	0	0	0	0	0	0	0	0	0	1	1	6.66666667	81	0	0	129
AMOXAPINE	0	0	1	0	0	0	0	0	0	0	0	0	0	0	0	0	0	1	1	6.66666667	81	0	0	129
VALSARTAN	0	0	1	0	0	0	0	0	0	0	0	0	0	0	0	0	0	1	1	6.66666667	81	0	0	129
LOSARTAN	0	0	1	0	0	0	0	0	0	0	0	0	0	0	0	0	0	1	1	6.66666667	81	0	0	129
TELMISARTAN	0	0	1	0	0	0	0	0	0	0	0	0	0	0	0	0	0	1	1	6.66666667	81	0	0	129
BOSENTAN	0	0	1	0	0	0	0	0	0	0	0	0	0	0	0	0	0	1	1	6.66666667	81	0	0	129
MACITENTAN	0	0	1	0	0	0	0	0	0	0	0	0	0	0	0	0	0	1	1	6.66666667	81	0	0	129
DIPHENHYDRAMINE	0	0	1	0	0	0	0	0	0	0	0	0	0	0	0	0	0	1	1	6.66666667	81	0	0	129
CETIRIZINE	0	0	1	0	0	0	0	0	0	0	0	0	0	0	0	0	0	1	1	6.66666667	81	0	0	129
FEXOFENADINE	0	0	1	0	0	0	0	0	0	0	0	0	0	0	0	0	0	1	1	6.66666667	81	0	0	129
EPINASTINE	0	0	1	0	0	0	0	0	0	0	0	0	0	0	0	0	0	1	1	6.66666667	81	0	0	129
OLOPATADINE	0	0	1	0	0	0	0	0	0	0	0	0	0	0	0	0	0	1	1	6.66666667	81	0	0	129
DIBENZEPIN	0	0	1	0	0	0	0	0	0	0	0	0	0	0	0	0	0	1	1	6.66666667	81	0	0	129
AZELASTINE	0	0	1	0	0	0	0	0	0	0	0	0	0	0	0	0	0	1	1	6.66666667	81	0	0	129
TRIPROLIDINE	0	0	1	0	0	0	0	0	0	0	0	0	0	0	0	0	0	1	1	6.66666667	81	0	0	129
DESLORATADINE	0	0	1	0	0	0	0	0	0	0	0	0	0	0	0	0	0	1	1	6.66666667	81	0	0	129
RANITIDINE	0	0	1	0	0	0	0	0	0	0	0	0	0	0	0	0	0	1	1	6.66666667	81	0	0	129
FAMOTIDINE	0	0	1	0	0	0	0	0	0	0	0	0	0	0	0	0	0	1	1	6.66666667	81	0	0	129
NIZATIDINE	0	0	1	0	0	0	0	0	0	0	0	0	0	0	0	0	0	1	1	6.66666667	81	0	0	129
CIMETIDINE	0	0	1	0	0	0	0	0	0	0	0	0	0	0	0	0	0	1	1	6.66666667	81	0	0	129
TOLAZOLINE	0	0	1	0	0	0	0	0	0	0	0	0	0	0	0	0	0	1	1	6.66666667	81	0	0	129
HALOPERIDOL	0	0	1	0	0	0	0	0	0	0	0	0	0	0	0	0	0	1	1	6.66666667	81	0	0	129
RISPERIDONE	0	0	1	0	0	0	0	0	0	0	0	0	0	0	0	0	0	1	1	6.66666667	81	0	0	129
CLOZAPINE	0	0	1	0	0	0	0	0	0	0	0	0	0	0	0	0	0	1	1	6.66666667	81	0	0	129
OLANZAPINE	0	0	1	0	0	0	0	0	0	0	0	0	0	0	0	0	0	1	1	6.66666667	81	0	0	129
QUETIAPINE	0	0	1	0	0	0	0	0	0	0	0	0	0	0	0	0	0	1	1	6.66666667	81	0	0	129
AMISULPRIDE	0	0	1	0	0	0	0	0	0	0	0	0	0	0	0	0	0	1	1	6.66666667	81	0	0	129
METHYSERGIDE	0	0	1	0	0	0	0	0	0	0	0	0	0	0	0	0	0	1	1	6.66666667	81	0	0	129
DEXFENFLURAMINE	0	0	1	0	0	0	0	0	0	0	0	0	0	0	0	0	0	1	1	6.66666667	81	0	0	129
MIRTAZAPINE	0	0	1	0	0	0	0	0	0	0	0	0	0	0	0	0	0	1	1	6.66666667	81	0	0	129
METOCLOPRAMIDE	0	0	1	0	0	0	0	0	0	0	0	0	0	0	0	0	0	1	1	6.66666667	81	0	0	129
PRUCALOPRIDE	0	0	1	0	0	0	0	0	0	0	0	0	0	0	0	0	0	1	1	6.66666667	81	0	0	129
LUTROPIN ALFA	0	0	1	0	0	0	0	0	0	0	0	0	0	0	0	0	0	1	1	6.66666667	81	0	0	129
MENOTROPINS	0	0	1	0	0	0	0	0	0	0	0	0	0	0	0	0	0	1	1	6.66666667	81	0	0	129
GONADOTROPIN, CHORIONIC	0	0	1	0	0	0	0	0	0	0	0	0	0	0	0	0	0	1	1	6.66666667	81	0	0	129
OXYTOCIN	0	0	1	0	0	0	0	0	0	0	0	0	0	0	0	0	0	1	1	6.66666667	81	0	0	129
CARBETOCIN	0	0	1	0	0	0	0	0	0	0	0	0	0	0	0	0	0	1	1	6.66666667	81	0	0	129
ATOSIBAN	0	0	1	0	0	0	0	0	0	0	0	0	0	0	0	0	0	1	1	6.66666667	81	0	0	129
DESMOPRESSIN	0	0	1	0	0	0	0	0	0	0	0	0	0	0	0	0	0	1	1	6.66666667	81	0	0	129
CONIVAPTAN	0	0	1	0	0	0	0	0	0	0	0	0	0	0	0	0	0	1	1	6.66666667	81	0	0	129
VASOPRESSIN	0	0	1	0	0	0	0	0	0	0	0	0	0	0	0	0	0	1	1	6.66666667	81	0	0	129
ALPROSTADIL	0	0	1	0	0	0	0	0	0	0	0	0	0	0	0	0	0	1	1	6.66666667	81	0	0	129
MISOPROSTOL	0	0	1	0	0	0	0	0	0	0	0	0	0	0	0	0	0	1	1	6.66666667	81	0	0	129
LATANOPROST	0	0	1	0	0	0	0	0	0	0	0	0	0	0	0	0	0	1	1	6.66666667	81	0	0	129
TRAVOPROST	0	0	1	0	0	0	0	0	0	0	0	0	0	0	0	0	0	1	1	6.66666667	81	0	0	129
BIMATOPROST	0	0	1	0	0	0	0	0	0	0	0	0	0	0	0	0	0	1	1	6.66666667	81	0	0	129
IBODUTANT	0	0	1	0	0	0	0	0	0	0	0	0	0	0	0	0	0	1	1	6.66666667	81	0	0	129
FOSAPREPITANT	0	0	1	0	0	0	0	0	0	0	0	0	0	0	0	0	0	1	1	6.66666667	81	0	0	129
APREPITANT	0	0	1	0	0	0	0	0	0	0	0	0	0	0	0	0	0	1	1	6.66666667	81	0	0	129
NETUPITANT	0	0	1	0	0	0	0	0	0	0	0	0	0	0	0	0	0	1	1	6.66666667	81	0	0	129
FOSNETUPITANT	0	0	1	0	0	0	0	0	0	0	0	0	0	0	0	0	0	1	1	6.66666667	81	0	0	129
SERATRODAST	0	0	1	0	0	0	0	0	0	0	0	0	0	0	0	0	0	1	1	6.66666667	81	0	0	129

**KEY: 1 = Drug predicted to be relevant in comparison; 0 = drug NOT predicted to be relevant in comparison

We then analyzed the drugs that were identified as having targets in pathways affected by SARS-CoV-2 to determine which were predicted to reverse the effects of the viral infection on the affected pathway. Of the 42 drugs that targeted SARS-CoV-2 related pathways, 27 were predicted to reverse viral effects on these pathways. We then performed a literature search to determine if any of these drugs had been previously used to treat COVID-19 or had been identified as potential therapeutics by other research groups. We found six of the 12 therapeutics that we predicted to be useful against SARS-CoV-2 had already shown positive results in clinical tests including canakinumab, anakinra, tocilizumab, sarilumab, and baricitinib. These results give further support to the validity of our computational workflow.

## Discussion

The computational workflow that we describe in this work predicts human therapeutic targets from signaling pathways and gene expression that are significantly affected during infection. We applied this workflow within the context of a meta-analysis that consisted of multiple public transcriptomic datasets of Betacoronaviruses. We then validated our results by comparing our predictions against recently published studies reporting therapeutics for SARS-CoV-2. Specifically, our downstream analyses enable us to calculate significant signaling pathways from DE genes using the SPIA algorithm as well as to predict potential therapeutics and their respective targets. Our analysis revealed thousands of DE genes, 580 enriched functional terms, as well as 249 significant pathways, including 38 pathways that were specifically affected during infection with SARS-CoV-2. It is important to point out that this workflow focuses on identifying human drug targets for two reasons: 1) to aid in the repurposing of existing drugs against emerging pathogens, and 2) to reduce the likelihood that a pathogen will develop resistance against the therapeutic(s) since they interact with a human protein that is much less likely to mutate than a viral protein.

Our approach differs from prior meta-analyses by focusing on a consistent, robust ARMOR-based RNA-seq preprocessing workflow for all datasets as well as a downstream pathway perturbation analysis. Previous studies have used a variety of approaches to predict possible therapeutics ^42– 46^, but none have combined the various aspects that are described in this work. The SPIA algorithm we used in this study has been shown to provide robust statistical results of perturbed pathways while not simply enriching for DE genes ^34^. It also enabled us to identify protein components in signaling pathways that could be reversed to reduce the adverse signs and symptoms that occur during infection, which differs from simply attempting to target DE genes. This approach drastically differs from some attempts to directly target DE genes without accounting for how a treatment may affect the cellular protein-protein interaction network. In this analysis we have compared significant DE genes and pathways identified in multiple different studies that used different MOIs, timepoints, and cell types. We found many DE genes and pathways that were affected across a variety of samples which increases our confidence in our results as those genes and pathways appear to be affected more by the virus itself than by other variables such as cell type, MOI, or timepoint.

Although our approach was dependent on identifying DE genes using a consistent preprocessing workflow, the focus of our analysis was to identify relevant functions, pathways, and potential drug targets from the DE genes. These processed data could then be used to better understand the underlying biological mechanisms of pathogenesis, but to better identify host-based therapeutic targets. A subset of the enriched annotations identified by the Camera algorithm have been reported to be relevant during infection with SARS-CoV-2 in clinical studies including “response to chemokine” ^47^, “humoral immune response” ^48– 50^, “chronic inflammatory response” ^51, 52^, “toll like receptor binding” ^53, 54^, “interleukin-6 production” ^55^ and citrate metabolism. A separate study of COVID-19 identified Bradykinin as potentially playing a role in pathogenesis ^56^. Interestingly, the annotations for this gene and its receptor include several of the enriched terms we identified such as arachidonic acid, inflammation, and G-protein coupled receptor activity. We interpret these separate studies to validate the findings of our functional enrichment analysis and anticipate that future studies will shed additional insight into the underlying mechanism(s) of viral pathogenesis.

Many of the signaling pathway components that we identified in this study have also been reported previously. One prior proteomics study reported applying translation inhibitors to Caco-2 cells infected with SARS-CoV-2 reduced virus replication ^57^, which supports our pathway perturbation results. Other studies have reported that the ORF6, ORF8, and nucleocapsid proteins of SARS-CoV-2 are antagonists of type-I interferon and NF-kB in HEK-293 T cells ^58^ or the induction of apoptosis during viral infection ^59^. In contrast, our meta-analysis predicted the type-I interferon pathway to be activated, suggesting either that this response could be dependent on the cell type, or that a potential redundant mechanism in the host cell can still turn on this pathway even if certain components are down-regulated. Our analysis predicted that noncanonical NF-kB signaling was inhibited during MERS and SARS infection, while being activated during SARS-CoV-2 infection in Calu-3 cells. While it is possible that this difference is due to a cell-specific response from the studies included in our meta-analysis, a NF-kB inhibitor applied to Vero E6 cells infected with SARS-CoV-2 has been shown to reduce cytopathic effects and virus plaques ^60^. This result suggests that NF-kB signaling may be active and contribute to the inflammatory signs and symptoms observed during virus infection, which agrees with our results. The Toll-like receptor and JAK-STAT pathways were previously found to be relevant to SARS-CoV-2 infection in A549 cells, which we also identified in infected Calu-3 cells ^42^. Citrate metabolism was also identified as an important pathway by our analysis and has been supported elsewhere ^61^.

Although the US FDA has only issued emergency use authorization for therapeutic treatment for severe cases of COVID-19, a multitude of studies have reported results from attempting to treat patients with a variety of existing FDA approved therapeutics ^62– 69^. We found that 27 of our 42 predicted therapeutics are predicted to “reverse” the effect on the pathways relevant to the viral infections being compared. Twelve of the 27 drugs that were predicted to be potential therapeutics against SARS-CoV-2 and are used to combat autoimmune or inflammatory diseases such as MS, SLE, and rheumatoid arthritis while others have been used in cancer treatments and against viral infections such as hepatitis C and human immunodeficiency virus. Six of these 12 drugs have been used to treat COVID-19 in patients including canakinumab, anakinra, tocilizumab, sarilumab, baricitinib, and hydroxychloroquine ^70^. Two other drugs, maraviroc and brodalumab, have been identified as potential treatments via cell cultures and computer models ^71– 74^. Others on the list such as benralizumab have been identified through anecdotal data as biologics that potentially exert a prophylactic effect for SARS-CoV-2, when they are taken at the time of infection ^75^. Baricitinib is of particular interest as the US FDA has issued emergency use authorization for its use in conjunction with remdesivir in the treatment of COVID-19 patients over the age of two that have been hospitalized and require supplemental oxygen, invasive mechanical ventilation, or extracorporeal membrane oxygenation ^76^. Baricitinib has also been shown to be effective against COVID-19 when combined with corticosteroids ^77^. A small study involving Tocilizumab has also shown it can be useful in improving the outcome of patients with severe COVID-19 ^78^.

## Conclusions

In conclusion, we developed and applied an important bioinformatics workflow, that combines existing tools with custom scripts, to predict potential human therapeutic targets. This workflow was then validated through a meta-analysis of publicly available transcriptomics data. The multiple Betacoronavirus and SARS-CoV-2 datasets revealed significant genes, annotations, signaling pathways, and human proteins that could be targeted by therapeutics during infection with various Betacoronaviruses. It is important to recognize that many of the predictions made by our workflow have been supported by experimental and clinical work on this virus, which suggests that our approach could enable the rapid identification of relevant therapeutics against future emerging pathogens.

## Abbreviations

SARS-CoV-2: Severe acute respiratory syndrome coronavirus-2

COVID-19: Coronavirus disease-2019

GO: Gene ontology

SARS / SARS-CoV: Severe acute respiratory syndrome coronavirus

MERS / MERS-CoV: Middle east respiratory syndrome coronavirus

ACE2: Angiotensin-converting enzyme 2

FDA: Food and Drug Administration

GEO: Gene expression omnibus

NCBI: National center for biotechnology information

ARMOR: Automated Reproducible MOdular Workflow for Preprocessing and Differential Analysis of RNA-seq Data

SPIA: Signaling pathway impact analysis

DE: Differentially expressed

FDR: False-discovery rate

## Data availability

### Underlying data

GEO: Transcriptomic analysis of MERS-CoV infected Calu-3 cell with or without AM580 treatment, Accession number GSE122876: https://www.ncbi.nlm.nih.gov/geo/query/acc.cgi?acc=GSE122876

GEO: Transcriptomic analysis of the Novel Middle East Respiratory Syndrome Coronavirus (Human, MRC5 cells, Accession number GSE56192: https://www.ncbi.nlm.nih.gov/geo/query/acc.cgi?acc=GSE56192

GEO: Transcriptional response to SARS-CoV-2 infection, Accession number GSE147507: https://www.ncbi.nlm.nih.gov/geo/query/acc.cgi?acc=GSE147507

GEO: Transcriptomic Analysis Of circRNAs/miRNAs/mRNAs upon Middle East Respiratory Syndrome Coronavirus (MERS-CoV) infection, Accession number GSE139516: https://www.ncbi.nlm.nih.gov/geo/query/acc.cgi?acc=GSE139516

Code for this workflow can be found on GitHub: https://github.com/bpickett/Pathway2Targets.

Archived code as at time of publication: http://doi.org/10.5281/zenodo.4706197 ^79^.

Code is available under the terms of the Creative Commons Zero "No rights reserved" data waiver (CC0 1.0 Public domain dedication).
