# Supplementary material for: A signaling pathway-driven bioinformatics pipeline for predicting therapeutics against emerging infectious diseases
Source: F1000Res. 2021 Aug 20;10:330. Originally published 2021 Apr 29. [Version 2] doi: 10.12688/f1000research.52412.2 (PMC8607308; doi:10.12688/f1000research.52412.2)
Supplement: Peer review history

# Peer review: Reviewer response for version 2

**DOI:** 10.5256/f1000research.76722.r99992

**Published:** 2021-11-19

**Authors:** Soumya Lipsa Rath

**Affiliations:** 1 National Institute of Technology, Warangal, Telangana, India

The manuscript by Pickett *et al.* is very well presented. The authors have systematically analysed the RNA-sequencing data of SARS, SARS-COV-2 and MERS coronaviruses. They narrowed down to a subset of pathways that are commonly affected and predicted the existing FDA approved drugs that could be used to treat COVID-19. They have designed a pipeline on how one could quickly investigate if the existing drugs could be repurposed for a disease. The reviewer feels the work would be of potential interest to the readers and should be accepted for indexing.

Is the rationale for developing the new method (or application) clearly explained?

Yes

Is the description of the method technically sound?

Yes

Are the conclusions about the method and its performance adequately supported by the findings presented in the article?

Yes

If any results are presented, are all the source data underlying the results available to ensure full reproducibility?

Yes

Are sufficient details provided to allow replication of the method development and its use by others?

Yes

Reviewer Expertise:

Structural Bioinformatics, Computational Biophysics, MD Simulation

I confirm that I have read this submission and believe that I have an appropriate level of expertise to confirm that it is of an acceptable scientific standard.

# Peer review: Reviewer response for version 1

**DOI:** 10.5256/f1000research.55686.r89746

**Published:** 2021-08-02

**Authors:** Babatunde Joseph Oso

**Affiliations:** 1 Department of Biochemistry, McPherson University, Seriki Sotayo, Nigeria

The authors presented an original article on the signaling pathway-driven bioinformatics pipeline for predicting therapeutics against emerging infectious diseases. The authors are commended for the clarity and straight-forward style of the article. However, this manuscript needs to be improved regarding the following aspects:

- It would be interesting if a bibliographic review on the importance of bioinformatics workflow and meta-analysis vis-à-vis prediction of potential human therapeutics can be included in the introduction section.
- The argument presented in the introduction section should include a clear description of the statement problem.
- Generally, the sentences should be well structured for a better flow and limit the use of possessive pronouns.

Is the rationale for developing the new method (or application) clearly explained?

Yes

Is the description of the method technically sound?

Yes

Are the conclusions about the method and its performance adequately supported by the findings presented in the article?

Yes

If any results are presented, are all the source data underlying the results available to ensure full reproducibility?

Yes

Are sufficient details provided to allow replication of the method development and its use by others?

Yes

Reviewer Expertise:

Biochemistry

I confirm that I have read this submission and believe that I have an appropriate level of expertise to confirm that it is of an acceptable scientific standard.

# Peer review: Reviewer response for version 1

**DOI:** 10.5256/f1000research.55686.r85467

**Published:** 2021-06-02

**Authors:** José Pedro Cerón-Carrasco

**Affiliations:** 1 Bioinformatics and High Performance Computing Research Group (BIO-HPC), Faculty of Polytechnic Science, UCAM Catholic University of Murcia, Murcia, Spain

I feel this work touches up an alternative approach to the more classical computational protocol of performing virtual screening by docking methods + molecular dynamics to refine binding energies.

 Herein, the proposed method uses RNA-sequencing data with a focus on defining differentially expressed genes, enriched Gene Ontology (GO) terms, and dysregulated pathways in infected cells.

 On the brightest side, that theoretical framework allows for a simultaneous search of small molecules, antibody and protein. The authors stated that their predictions yield to a final list of 42 therapeutics. 27 of such drugs have been reported to exhort an effect against the virus. It remains unrevealed if these other 15 are negative positive or pending possible treatments. A more 'experimental' reader might find a next candidate in that list.

Is the rationale for developing the new method (or application) clearly explained?

Yes

Is the description of the method technically sound?

Yes

Are the conclusions about the method and its performance adequately supported by the findings presented in the article?

Yes

If any results are presented, are all the source data underlying the results available to ensure full reproducibility?

Yes

Are sufficient details provided to allow replication of the method development and its use by others?

Yes

Reviewer Expertise:

Computational Chemistry for Drug Design.

I confirm that I have read this submission and believe that I have an appropriate level of expertise to confirm that it is of an acceptable scientific standard, however I have significant reservations, as outlined above.

# Response

**Published:** 2021-08-17

**Authors:** Brett Pickett

**Affiliations:** Brigham Young University, USA

We thank the reviewer for their helpful comment and agree that researchers would could indeed make good use of the complete list of drugs that we predicted to be useful against SARS-CoV-2. To address this comment we will update Table 4 in the next manuscript version to now include not only all of the predicted drugs, but also summaries of how each drug ranks across all analyzed Coronavirus datasets and also across all SARS-CoV-2 datasets. We believe that this change will improve the readability and relevance of our study.
